# Clinical Trial on the Characteristics of Zheng Classification of Pulmonary Diseases Based on Infrared Thermal Imaging Technology

**DOI:** 10.1155/2013/218909

**Published:** 2013-03-31

**Authors:** Jin-xia Ni, Si-hua Gao, Yu-hang Li, Shi-lei Ma, Tian Tian, Fang-fang Mo, Liu-qing Wang, Wen-zeng Zhu

**Affiliations:** ^1^Dongzhimen Hospital Affiliated to Beijing University of Chinese Medicine, Beijing 100700, China; ^2^Beijing University of Chinese Medicine, Beijing 100029, China; ^3^China Academy of Chinese Medicine Sciences, Beijing 100029, China; ^4^Guang'anmen Hospital, China Academy of Chinese Medicine Sciences, Beijing 100053, China

## Abstract

Zheng classification study based on infrared thermal imaging technology has not been reported before. To detect the relative temperature of viscera and bowels of different syndromes patients with pulmonary disease and to summarize the characteristics of different Zheng classifications, the infrared thermal imaging technology was used in the clinical trial. The results showed that the infrared thermal images characteristics of different Zheng classifications of pulmonary disease were distinctly different. The influence on viscera and bowels was deeper in phlegm-heat obstructing lung syndrome group than in cold-phlegm obstructing lung syndrome group. It is helpful to diagnose Zheng classification and to improve the diagnosis rate by analyzing the infrared thermal images of patients. The application of infrared thermal imaging technology provided objective measures for medical diagnosis and treatment in the field of Zheng studies and provided a new methodology for Zheng classification.

## 1. Introduction

Infrared thermal imaging technology is a noninvasive imaging procedure used to record the thermal patterns using infrared camera. It is widely applied in the field of biomedicine. It is believed that infrared thermal imaging cameras can potentially be used to detect subjects with fever, the cardinal symptom of SARS, and avian influenza [1]. And it has been previously utilized in the detection of breast cancer [2], in the diagnosis and management of erectile dysfunction [3], and in the evaluation of tear evaporation from ocular surface [4, 5]. Regarded as a speedy, comprehensive, convenient, and fairly accurate means, the use of infrared thermal imaging technology offers great opportunities for the study of Zheng classification of pulmonary diseases. While the relative temperature of viscera and bowels is detected by infrared camera, the function of viscera and bowels is visualized. Previous study showed the temperature of viscera and bowels regulated in a special rule under physiological conditions [6]. Would the relative temperature of viscera and bowels of patients with pulmonary diseases from different Zheng groups change while compared with healthy people? How about the characteristics of Zheng classification of pulmonary diseases based on infrared thermal imaging technology? With these questions, we carried out these relative clinical trials.

## 2. Methods

### 2.1. Selection of Study Participants

72 healthy volunteers (group A) were enrolled from community resident in Dongcheng District of Beijing. 111 patients with pulmonary disease including chronic obstructive pulmonary disease, bronchial asthma, and chronic bronchitis were derived from the outpatient of Dongzhimen Hospital Affiliated to Beijing University of Chinese Medicine. Among these patients, 35 cases were diagnosed cold-phlegm obstructing lung syndrome (Group B) and 76 cases were diagnosed phlegm-heat obstructing the lung syndrome (Group C). The clinical trial was approved by the Ethics Committee of Dongzhimen Hospital Affiliated to Beijing University of Chinese Medicine. The gender and age distribution was shown in Tables 1 and 2.

### 2.2. Diagnosis Criteria of Chronic Obstructive Pulmonary Disease

Reference to the *practical internal medicine* and *chronic obstructive pulmonary disease treatment guidelines (2007 revision)* was written by chronic obstructive pulmonary disease study group of Chinese Society of Respiratory Diseases [7].

### 2.3. Diagnosis Criteria of Bronchial Asthma

Reference to the *Bronchial asthma prevention and treatment guidelines* was revised by Asthma study group of Chinese Society of Respiratory Diseases on November 7, 2002 [8].

### 2.4. Diagnosis Criteria of Chronic Bronchitis

Reference to the *Chronic bronchitis clinical diagnosis and the efficacy criteria was* revised by National Clinical Professional meeting of chronic bronchitis in 1979 [9].

### 2.5. Diagnosis Criteria of Zheng Classification of Pulmonary Diseases

Reference to the *Chinese internal medicine* and *TCM Zheng Diagnosis and efficacy standard* [10] and *TCM terms* [11].

### 2.6. Diagnosis Criteria of Phlegm-Heat Obstructing Lung Syndrome

Primary symptoms include (1) cough or (and) dyspnea; (2) yellow sticky sputum.

Associated symptoms include (1) vexation, (2) abdominal distention and fullness, (3) dry mouth and thirst and like drinking cold, (4) fever but without aversion to cold, (5) yellow urine, (6) dyschezia, (7) red tongue, (8) yellow or yellow with greasy tongue fur, and (9) slippery and rapid pulse.

### 2.7. Diagnosis Criteria of Cold-Phlegm Obstructing Lung Syndrome

Primary symptoms: (1) cough or (and) dyspnea (2) white sputum.

Associated symptoms include (1) oppression in the chest, (2) fear of cold and cold extremities, (3) pale tongue, (4) white with lubricating tongue fur, (5) string-like with tight pulse.

Patient with primary symptoms and two associated symptoms can be diagnosed.

### 2.8. Selection Criteria to Patients with Pulmonary Diseases

1. In line with the diagnosis criteria of chronic obstructive pulmonary disease or bronchial asthma or chronic bronchitis.
2. In line with the diagnosis criteria of phlegm-heat obstructing the lung syndrome or cold-phlegm obstructing lung syndrome.
3. 46 to 75 years old.
4. Without skin diseases or skin allergies or skin surface damage.
5. Patients in stable condition can coordinate with the researchers to completed the trial independently.
6. Signed informed consent.

### 2.9. Method of Infrared Thermal Imaging Detection

ATIR-M301 medical infrared thermal imager (produced by Chongqing Wei-Lian company with 0.05°C temperature resolution and 3 mrad spatial resolution) was used in this clinical trial. The scanning room temperature was controlled at 24 ± 2°C and the humidity was 65%–70%. In order to make the surface temperature balanced, the healthy volunteer or patients exposed completely the body and stood quietly in the scanning room for 15 minutes to adapt to the environmental temperature before being checked. The anteroposterior images were scanned and saved to be analyzed. Medical thermography analysis software was used to detect and analyze the relative temperature of viscera and bowels of healthy people and patients with different Zheng of pulmonary diseases. The relative temperature was measured in the region that viscera and bowels project.

### 2.10. Statistical Analysis

*χ* ^2^ test was used to analyze the enumeration data. One-way ANOVA was used to analyze the data between the normal and different Zheng classification groups. *q* test and LSD method were used for the paired comparisons, with *P* < 0.05 denoted as significant.

## 3. Results

### 3.1. The Relative Temperature Characteristics of Viscera and Bowels in Different Groups

**Figure 1:**
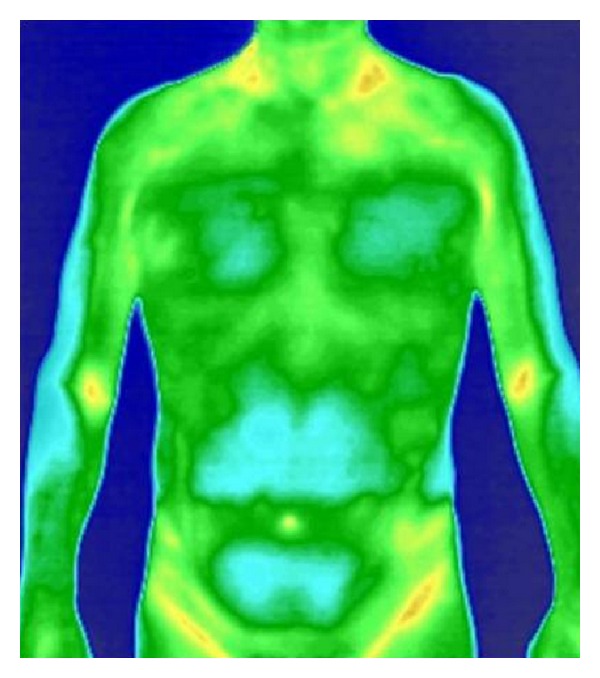
The infrared thermal images of healthy people.

**Figure 2:**
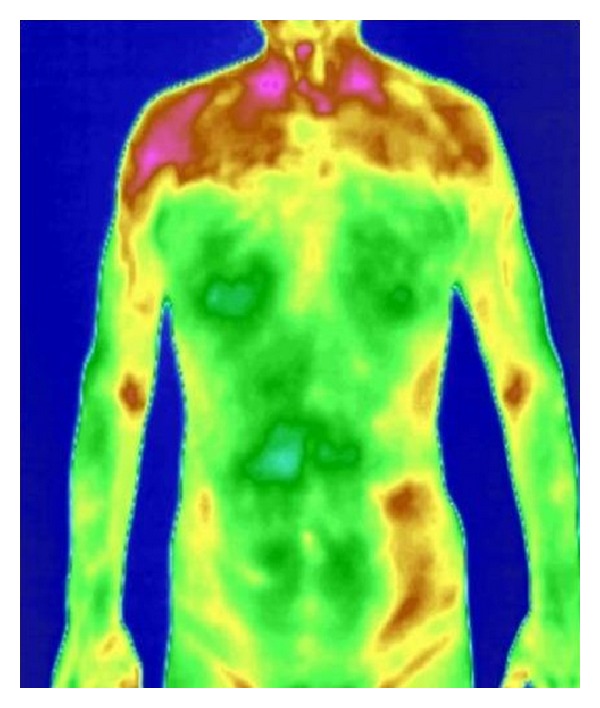
The infrared thermal images of patient with phlegm-heat obstructing lung syndrome.

**Figure 3:**
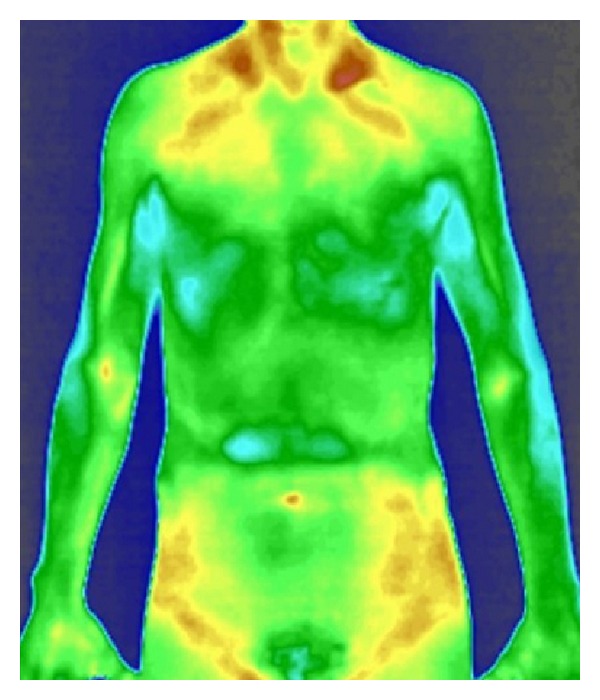
The infrared thermal images of patient with cold-phlegm obstructing lung syndrome.

The trial showed that in the normal group and in the cold-phlegm obstructing lung syndrome group, as well as in the phlegm-heat obstructing lung syndrome group, the maximum temperature of viscera and bowels was taken in the lung region, followed by the lower temperature in both the descending colon and the ascending colon regions; see Figures 1, 2, and 3.

### 3.2. The Comparison of Relative Temperature of Viscera and Bowels among Different Groups

The results showed that the temperature characteristics of viscera and bowels were different in different Zheng classifications of pulmonary diseases. Compared with normal group, the relative temperature of lung, descending colon, ascending colon, small intestine, and stomach significantly increased in the phlegm-heat obstructing lung syndrome group, and there was a statistical difference. While compared with normal group, the relative temperature of lung and descending colon was significantly increased in the cold-phlegm obstructing lung syndrome group, and there was a statistical difference, but the relative temperature of ascending colon, small intestine, and stomach was not significantly different. Compared with cold-phlegm obstructing lung syndrome group, the relative temperature of lung and small intestine significantly increased in phlegm-heat obstructing lung syndrome group, and there was a statistical difference. See Tables 3 and 4.

## 4. Discussion

Syndrome differentiation is one of the important guidance and principles in the clinic of traditional Chinese medicine. Zheng classification is the basis of syndrome differentiation, and it is a method to distinguish between the health and disease by correlation of all four examinations. The research on Zheng classification is one of the cut-in points on integration of traditional Chinese and Western medicine [12]. It is an important way to classify diseases with syndrome differentiation and a mutual supplement for integration of traditional Chinese and Western medicine, also an important measure and approach for developing Chinese medicine and innovating biomedicine. It is meaningful in both theoretical guidance and clinical practice [13].

Epidemiological studies have shown that the main leading causes of death in China urban population in 2002-2003 were cancer and heart diseases, respectively; the death due to respiratory diseases accounted for 15.63% of the total mortality year round. In 2006, the death due to respiratory diseases was in the fourth place [14]. The four leading causes of death in all scenarios were projected to be ischaemic heart disease, cerebrovascular disease (stroke), HIV/AIDS, and COPD [15]. Thereby, the research on Zheng classification of pulmonary diseases is of great significance in guiding clinical practice. Although in the 1980s a TCM syndromes standard was studied, but the name of Zheng, the Zheng classification and the diagnosis of Zheng are not yet unified [16]. It is practical that the disease classification should be a reference frame and the research on Zheng classification should be carried out on the basis of the disease classification [12]. In this clinical trial, the common pulmonary diseases including chronic obstructive pulmonary disease, bronchial asthma, and chronic bronchitis were referenced as frame and the research on Zheng classification was studied.

Compared with previous Zheng classification study, the infrared thermal imaging technology was used in this trial. The two opposing Zheng classification including cold and heat of pulmonary disease were studied to distinguish the characteristics by infrared thermal imaging.

Infrared thermal imaging technology is a functional imaging technique. The body temperature can be continuously traced objectively and accurately by medical infrared imaging at any point and two-dimensional temperature field. Infrared thermal imaging provides visual and diversified information of body temperature distribution marked with different colors to the clinicians. It provided an important way for disease diagnosis by analyzing the heat map and the difference of surface temperature [17]. In many diseases the blood flow varied and influenced the skin temperature. IR imaging offers a useful and noninvasive approach to disease diagnosis and treatment (as therapeutic aids), in particular in the areas of rheumatology, dermatology, orthopaedics, and circulatory abnormalities [18]. With the development of infrared thermal imaging technology, it was used not only in breast cancer screening and diagnosis [19], but also in the diagnosis of a variety of diseases, for example, for early diagnosis and study of variety of tumors [20–24], the diagnosis of breast disease [25–27], the study of peritoneal inflammatory diseases [28], early screening of myocardial ischemia [29], the study of the clinical efficacy of herbs and testing Chinese herbal nature [30, 31], the research of back acupoints thermal distribution on bronchial asthma patients [32], the thermographic observation of shoulder-hand syndrome following stroke [33], the study of the infrared radiant track along lung meridian of patients with lung diseases [34], the study of warm needling used in the treatment of lumbar disc herniation [35], and the study of early diagnosis of thyroid disease [36, 37]. It was reported that the far infrared thermogram was applied as an index for diagnosis of acute peripheral facial paralysis [38].

Zheng classification study based on infrared thermal imaging technology has not been reported before. Our previous studies showed that under physiological conditions, the relative temperature of viscera and bowels was regulated by a special rule and the relative temperature in descending order was ordered as follows: lung > descending colon > ascending colon > liver > stomach > kidney > small intestine [6]. In this trial, the relative temperature of viscera and bowels of patients with cold-phlegm obstructing lung or phlegm-heat obstructing lung was different from that of normal group, although the relative temperature of lung was highest, followed by lower temperature of descending colon and ascending colon. Compared with normal group, the relative temperature in the region of lung, descending colon, ascending colon, small intestine, and stomach was significantly increased in phlegm-heat obstructing lung syndrome group. While compared with normal group, the relative temperature in lung region and descending colon region was significantly increased in cold-phlegm obstructing lung syndrome group, but the relative temperature in the region of ascending colon, small intestine, and stomach was not significantly different. In the comparison between different Zheng classification, the relative temperature of lung and small intestine significantly increased in phlegm-heat obstructing lung syndrome group than in cold-phlegm obstructing lung syndrome group, and the difference was significant. Most of the viscera and bowels relative temperature in phlegm-heat obstructing lung syndrome group was higher than that in cold-phlegm obstructing lung syndrome group. The result indicated that phlegm-heat obstructing lung syndrome influenced viscera and bowels function deeper than cold-phlegm obstructing lung syndrome.

*Pairing of the viscera and bowels* is an important theory, which provides guidance to traditional Chinese medicine (TCM) clinical practice. The investigation has been the focus of research on the basic theory of TCM [39]. The results showed that the organs function was influenced to some degree when the physiological function of the lung is abnormal. From the point of view of infrared thermal imaging technology, the relative temperature of organs in different Zheng classification was not in the same level. But both in phlegm-heat obstructing lung syndrome group and in cold-phlegm obstructing lung syndrome group, the relative temperature of lung and descending colon was consistently influenced. It provided clinical evidence to the theory that *the lung and large intestine are exteriorly-interiorly related*. The application of infrared thermal imaging technology provided objective method for medical diagnosis and treatment in the field of Zheng studies and provided a new methodology for Zheng classification.

In the clinic, the traditional Chinese medicine Zheng classification of pulmonary diseases presented diversity. Experienced Chinese medicine practitioners usually make syndrome differentiation by four examinations obtained. But some Zheng classification diagnosed by some doctors were still not clear; they directly reduced the efficacy of treatment and even aggravated the patient's illness. In the clinic, Zheng classification diagnostic tools were lacking at present. The infrared thermal images characteristics of different Zheng classifications of pulmonary disease were distinctly different. The influence on viscera and bowels was deeper in phlegm-heat obstructing lung syndrome group than in cold-phlegm obstructing lung syndrome group. The viscera and bowels relative temperature of patients with different Zheng classification of pulmonary diseases was different. The trial indicated that it is helpful to diagnose Zheng classification and to improve the diagnosis rate by analyzing the infrared thermal images of patients.

## 5. Conclusion

The infrared thermal images characteristics of different Zheng classifications of pulmonary disease were distinctly different. The influence on viscera and bowels was deeper in phlegm-heat obstructing lung syndrome group than in cold-phlegm obstructing lung syndrome group. It is helpful to diagnose Zheng classification and to improve the diagnosis rate by analyzing the infrared thermal images of patients. The application of infrared thermal imaging technology provided objective measure for medical diagnosis and treatment in the field of Zheng studies and provided a new methodology for Zheng classification.

## Figures and Tables

**Table 1:** The distribution of gender in different groups (*n*).

Groups	*n *	Male	Female	*χ* ^2^	*P *
Group A	72	18	54	4.486	0.106
Group B	35	10	25		
Group C	76	31	45		

**Table 2:** The distribution of age range in different groups (*n*).

Groups	*n*	46~60 years old	61–75 years old	*χ* ^2^	*P*
Group A	72	53	19	4.728	0.094
Group B	35	19	16		
Group C	76	46	30		

**Table 3:** The temperature of ascending colon, descending colon, and small intestine compared with different groups (°C; $\overset{-}{X}\pm s$).

Groups	*n *	Ascending colon	Descending colon	Small intestine
Group A	72	31.09 ± 0.98	31.12 ± 0.92	30.26 ± 0.89
Group B	35	31.47 ± 1.08	31.60 ± 1.08^∆^	30.60 ± 1.03
Group C	76	31.77 ± 1.03^▲^	31.92 ± 1.00^▲^	31.03 ± 1.08^▲∗^

^∆^ *P*<0.05, ^▲^ *P* < 0.01 compared with group A; *P < 0.05 compared with group B.

**Table 4:** The temperature of stomach, lung, liver and kidney compared with different groups (°C; $\overset{-}{X}\pm s$).

Groups	*n *	Stomach	Lung	Liver	Kidney
Group A	72	30.34 ± 1.19	31.33 ± 0.98	30.63 ± 1.09	30.30 ± 1.04
group B	35	30.47 ± 1.16	31.73 ± 1.00^∆^	30.88 ± 1.15	30.49 ± 0.97
group C	76	30.94 ± 1.21^▲^	32.18 ± 0.95^▲∗^	30.98 ± 1.10	30.62 ± 1.02

^∆^ *P* < 0.05, ^▲^ *P* < 0.01 compared with group A; *P < 0.05 compared with group B.
